# Increased Presence of Antibodies against Type I Interferons and Human Endogenous Retrovirus W in Intensive Care Unit COVID-19 Patients

**DOI:** 10.1128/spectrum.01280-22

**Published:** 2022-07-19

**Authors:** Elena Rita Simula, Maria Antonietta Manca, Marta Noli, Somaye Jasemi, Stefano Ruberto, Sergio Uzzau, Salvatore Rubino, Pietro Manca, Leonardo A. Sechi

**Affiliations:** a Department of Biomedical Sciences, Division of Microbiology and Virology, University of Sassari, Sassari, Italy; b Struttura Complessa Microbiologia e Virologia, Azienda Ospedaliera Universitaria Sassari, Sassari, Italy; c Servizio Centro Trasfusionale, Azienda Ospedaliera Universitaria Sassari, Sassari, Italy; Johns Hopkins Hospital

**Keywords:** COVID-19, HERV-W, ICU, IFN-I, autoantibodies

## Abstract

In this work, we observed an increased presence of antibodies (Abs) against type I interferon (IFN-I) in coronavirus disease 2019 (COVID-19) patients admitted to the intensive care unit (ICU) compared to non-ICU COVID-19 patients and healthy control (HC) subjects. Human endogenous retrovirus W (HERV-W) can reactivate after viral infection; therefore, we also investigated the presence of antibodies against HERV-W envelope (HERV-W-env)-derived epitopes. A total of 113 subjects (41 female and 72 male subjects) were analyzed. A significant difference in autoantibodies against IFN-α, IFN-ω, and HERV-W was observed between HCs and ICU patients; indeed, the latter have higher levels of autoantibodies against IFN-α, IFN-ω, and HERV-W than subjects with mild COVID-19 and HCs. Neutralizing anti-IFN-I autoantibodies may affect the ability of IFN-I to bind to the type I interferon receptor (IFNAR), blocking the activation of the antiviral response.

**IMPORTANCE** In this work, we report the increased presence of IFN autoantibodies in correlation with HERV-W-env autoantibodies in ICU COVID-19 patients. The novelty of the results is in the association of these IFN autoantibodies with autoantibodies against HERV-W-env, a protein recently discovered to be overexpressed in lymphocytes of COVID-19 patients and correlated with severe disease and pneumonia. Type I IFNs are part of a complex cross-regulatory network; however, in a small percentage of cases, the increase in autoantibodies against these proteins may lead to damage to the host instead of protection against infectious diseases.

## INTRODUCTION

SARS-CoV-2 (severe acute respiratory syndrome coronavirus 2) is an enveloped, positive-sense, single-stranded RNA virus of the genus *Betacoronavirus* (1). It represents the causative agent of coronavirus disease 2019 (COVID-19), which escalated into a global pandemic in 2020 due to its rapid transmission. The first line of transmission is viral particles expelled from COVID-19-infected individuals as droplets (2). SARS-CoV-2 primarily infects and enters cells by the binding of the viral spike (S) protein with the host angiotensin-converting enzyme 2 (ACE2) receptor (3, 4).

When SARS-CoV-2 nucleic acid is recognized by the host, the antiviral defense starts to activate the synthesis of interferons (IFNs) triggered by a series of signaling cascades (5). This represents a strong antiviral defense that can interfere with each step of viral replication, representing the major first line of defense against viruses.

Among the three types of IFNs, type I IFN (IFN-I) is known for its effectiveness in limiting viral spread and its immunoregulatory role. The type I IFN family is a multigene cytokine family that encodes 13 partially homologous IFN-α subtypes in humans, a single IFN-β subtype, and several poorly defined single-gene products (IFN-ɛ, IFN-τ, IFN-κ, IFN-ω, IFN-δ, and IFN-ζ) (6). Overactivation of the IFN-I signaling pathway could lead to an uncontrolled inflammatory response, whereas a deficiency in IFN-I functions may involve a lack of host protection against virus infections. Autoantibodies (auto-Abs) that neutralize IFN-α and/or -ω were found in the plasma samples of patients with life-threatening COVID-19 pneumonia but not in asymptomatic or paucisymptomatic subjects (7).

Endogenous retroviral sequences have been integrated through repeated infections during evolution (8, 9). Indeed, about 8% of the human genome is composed of genetic elements arising from ancestral infection of germ cells during the last 100 million years by exogenous retroviruses, namely, human endogenous retroviruses (HERVs). An intriguing aspect of HERV evolution is coadaptation with the host. Notwithstanding, HERVs are normally silenced or expressed at low levels, and the adaptation process has allowed the development of new cellular functions (9).

Recently, human endogenous retrovirus W (HERV-W) has been associated with the severity of COVID-19 pathology. Unlike in healthy controls (HCs), higher expression levels of HERV-W envelope (HERV-W-env) have been observed in leukocytes of patients. Significantly, the percentage of HERV-W-env-positive lymphocytes was correlated with inflammatory markers and pneumonia severity in COVID-19 patients (8).

A protein encoded by HERV-W, syncytin-1, is involved in placental morphogenesis and regulates inflammatory and autoimmune responses (10). Recent studies have confirmed the involvement of HERVs in neurological diseases such as multiple sclerosis (MS) and amyotrophic lateral sclerosis (ALS). In particular, higher levels of Abs against HERV-K-env and HERV-W-env have been detected in the plasma samples of patients with ALS and MS, respectively, but not in healthy control subjects (11, 12).

The relationship between IFNs and endogenous retrovirus has been documented in different studies (13, 14). Recently, a positive correlation between IFN mRNA and HERV concentrations in children with COVID-19 has been documented. The potential role of type I and type II IFNs in upregulating HERV *trans*-activation is supported by the fact that the same correlation was not observed in uninfected children (15). In this study, we support the role of type I IFNs reported previously, finding an increased presence of Abs against type I interferons in patients admitted to the intensive care unit (ICU) among COVID-19 and HC subjects. Finally, we also investigated the possible link between the humoral response against HERV-W-env- and type I interferon-derived epitopes.

## RESULTS

**FIG 1:**
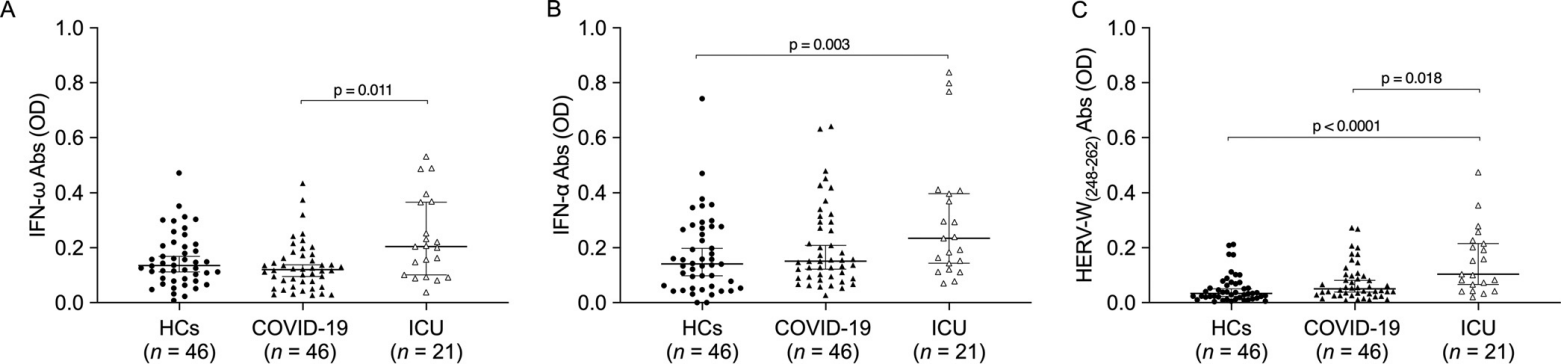
Analysis of humoral responses against IFN-ω (A)-, IFN-α (B)-, and HERV-W_(248–262)_ (C)-derived epitopes in the HC, COVID-19, and ICU groups. A Kruskal-Wallis test and Dunn’s *post hoc* analysis were performed. Scatterplots represent the medians with 95% confidence intervals (CIs), and the *P* value is indicated in the upper part of each graph. OD, optical density.

**FIG 2:**
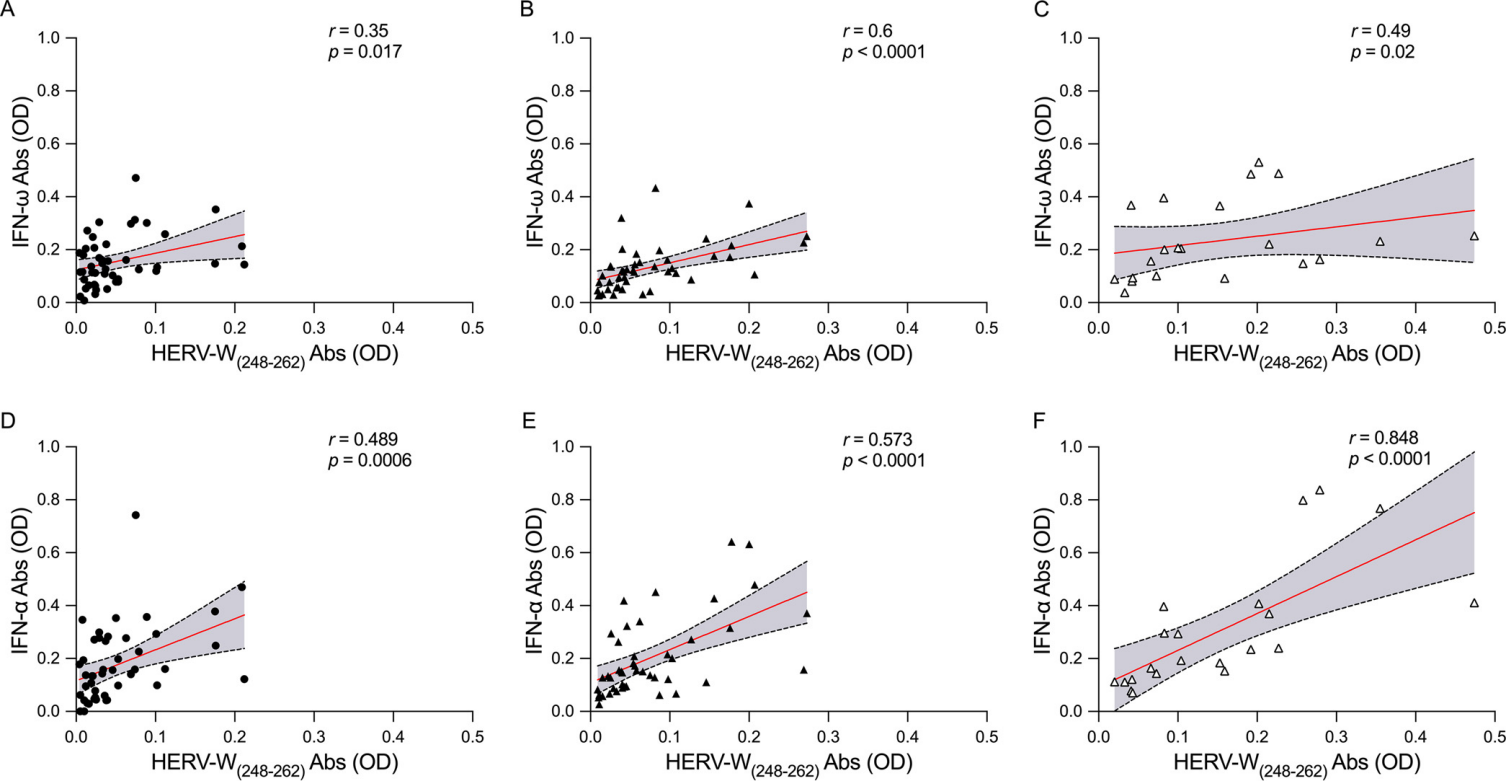
Scatterplots of humoral responses among HERV-W_(248–262)_- and IFN-derived epitopes in the HC and patient populations. The graphs show the correlations between HERV-W_(248–262)_ and IFN-ω in the HC (A), COVID-19 (B), and ICU (C) populations and the correlations between HERV-W_(248–262)_ and IFN-α in the HC (D), COVID-19 (E), and ICU (F) populations.

The Kruskal-Wallis test and Dunn’s *post hoc* analysis were performed to investigate the humoral response in plasma samples of HC subjects and COVID-19 and ICU patients matched by age and sex against the selected peptides shown in Table 1. A significant difference in Ab responses has been observed between HCs and ICU patients considering the presence of Abs against IFN-α peptide and the epitope derived from the envelope portion of HERV-W (HERV-W_(248–262)_), with *P* values of 0.003 and <0.0001, respectively (see Fig. 2B and C). The humoral responses between COVID-19 and ICU populations were significantly different, and statistically significant responses against IFN-ω and HERV-W_(248–262)_ were observed, with *P* values of 0.011 and 0.018, respectively (Fig. 1A and C).

**TABLE 1:** Epitopes identified in HERV-W-env, IFN-α, and IFN-ω

Epitope source	Epitope positions (aa)^*a*^	Epitope sequence
HERV-W-env_(248–262)_	248–262	NSQCIRWVTPPTQIV
IFN-α	103–119	GVGVTETPLMKEDSILA
IFN-ω	127–144	VGEGESAGAISSPALTLR

a aa, amino acids.

The relationship of the presence of HERV-W_(248–262)_ and IFN Abs was evaluated by Spearman’s correlation. The results showed a positive correlation in each study population. Briefly, as shown in Fig. 2A, in HC subjects, positive correlations were found for HERV-W_(248–262)_ and IFN-ω (*r *= 0.35; *P *= 0.017) and HERV-W_(248–262)_ and IFN-α (*r *= 0.489; *P *= 0.0006) (Fig. 2D); in COVID-19 patients, positive correlations were found for HERV-W_(248–262)_ and IFN-ω (*r *= 0.6; *P < *0.0001) (Fig. 2B) and HERV-W_(248–262)_ and IFN-α (*r *= 0.573; *P < *0.0001) (Fig. 2E); and in ICU patients, positive correlations were found for HERV-W_(248–262)_ and IFN-ω (*r *= 0.49; *P *= 0.02) (Fig. 2C) and HERV-W_(248–262)_ and IFN-α (*r *= 0.848; *P < *0.0001) (Fig. 2F).

## DISCUSSION

In this work, we report that ICU patients with life-threatening COVID-19 have higher levels of autoantibodies against IFN-α and IFN-ω than subjects with mild COVID-19 and HCs. Furthermore, ICU patients showed higher levels of Abs against HERV-W-env_(248–262)_. Neutralizing anti-IFN-I autoantibodies may act by affecting the ability of IFN-I to bind to the type I interferon receptor (IFNAR), therefore blocking the activation of the antiviral response (7, 16, 17).

The first evidence to state the relationship between autoantibodies against type I interferons and severe forms of COVID-19 disease comes from work published in 2020 (7). Those authors reported the presence of autoantibodies against type I interferons in at least 10% of patients with a life-threatening form of COVID-19 pneumonia (7). Later, the same group reported that nearly 4% of uninfected individuals over 70 years old presented neutralizing autoantibodies against type I interferons (16). The presence of these autoantibodies could account for nearly 20% of COVID-19 deaths, and the levels of neutralizing autoantibodies showed an increase with age (16). Additionally, Chang et al. reported autoantibodies against IFN-α2 in 45% of COVID-19 patients (17). Recent work by Manry et al. (18) proposed type I IFN autoantibodies as strong predictors of death in COVID-19 patients. Particularly, those authors evaluated the association between both the infection fatality rate (IFR) and the relative risk of death (RRD) and neutralizing autoantibodies against type I IFNs across age groups. Carriers of autoantibodies against both IFN-α and IFN-ω showed a higher RRD across age groups, particularly those less than 70 years old. These results further confirm the importance of type I IFN-neutralizing autoantibodies as predictors of life-threatening COVID-19 disease. The evidence collected about anti-IFN-I autoantibodies provides new insights into SARS-CoV-2 infection as well as the relationship with the severity of the disease. The presence of autoantibodies appears to be a distinctive feature of SARS-CoV-2 infection since numerous studies reported the presence of autoantibodies usually found in autoimmune diseases, such as vasculitis and rheumatic diseases, in patients with severe COVID-19 rather than in subjects with milder disease (19–21). The type I IFN defect in susceptibility to severe forms of COVID-19, discussed in light of the presence of Abs, provides a reasonable but partial explanation. Indeed, only assays and functional models could confirm the effects of these autoantibodies on inducing the defect observed in susceptibility to COVID-19. Intriguingly, the levels of Abs against the HERV-W-env_(248–262)_ epitope were remarkably higher in the ICU population than in the COVID-19 and HC populations and exhibited a strong correlation with anti-IFN-I autoantibodies, particularly anti-IFN-α.

To date, few studies have investigated the activation of HERV expression and autoantibodies in COVID-19 disease. It is well established that HERV expression can be triggered by different phenomena, such as infectious agents. Given the strong correlation found between HERV-W-env and IFN-α antibody levels (Fig. 2F) (*r* = 0.848; *P* < 0.0001), we suspect that SARS-CoV-2 induces the reactivation of HERV-W sequences, and thus, it may be considered responsible for the heightened adaptative response found in the ICU group.

The association between HERV-W-env and anti-IFN-α Abs has already been documented in a mouse animal model with anti-myelin oligodendrocyte glycoprotein (MOG) autoimmune responses, suggesting their potential relationship (22).

Indeed, HERV-W envelope was found to be highly expressed in T lymphocytes of COVID-19 patients, and the transcript levels were positively associated with clinical parameters and disease severity (8). Indirectly, in the present study, the increased presence of Abs against HERV-W-env supports its abnormal expression.

Furthermore, HERV-W envelope mRNA levels were strongly correlated with interleukin-6 (IL-6), IL-17, CXCL6, and monocyte chemoattractant protein 1 (MCP1). A recent study demonstrated positive correlations between the mRNA levels of HERVs and those of IFN-I and IFN-II in children with COVID-19 (15). Particularly, children affected by a severe form of COVID-19 presented decreased expression levels of the IFN-I and IFN-III genes along with reduced levels of HERV-W transcripts compared to those in children with mild symptoms. In summary, ICU patients with life-threatening COVID-19 show high levels of anti-IFN-I autoantibodies and HERV-W-env_(248–262)_ Abs compared to HCs and COVID-19 patients with mild/moderate disease. Additionally, the humoral responses against HERV-W-env_(248–262)_ and IFN-α are strongly correlated in ICU patients. Further investigation is required to deepen our understanding of the role of HERV-W in COVID-19 as well the impact of SARS-CoV-2 on HERV-W activation and the presence of autoantibodies against IFN-I. We can affirm that type I IFNs are part of a complex cross-regulatory network, which, in a small percentage of cases, leads to damage to the host instead of protection against infectious diseases (23). In addition, the levels of anti-IFN-I Abs should be evaluated in a population of individuals before and after SARS-CoV-2 infection in order to clarify the impact of SARS-CoV-2 on autoantibody levels, HERV activation, the clinical evolution of the disease, and the potential prognostic significance of these autoantibodies.

## MATERIALS AND METHODS

### Sample collection.

In this retrospective study, were enrolled a total of 113 subjects (41 female and 72 male subjects). The patient population included 46 COVID-19 subjects who had not manifested significant pathological symptoms (17 female and 29 male subjects; mean age ± standard deviation [SD], 66 ± 7.13 years) and 21 ICU patients (6 female and 15 male patients; mean age ± SD, 69 ± 7.26 years). Additionally, 46 sex-matched healthy controls (HCs) (18 female and 28 male subjects; mean age ± SD, 62 ± 4.18 years) were recruited at the Blood Transfusion Centre of Sassari. All methods for the investigation of plasma samples were approved by the ASL 1 Ethical ASL Committee of Sassari (2149/CE, 2015).

Peripheral blood was collected into K-EDTA tubes and was processed immediately after collection. Following centrifugation, the resulting supernatant was designated plasma. According to the protocol, plasma was immediately transferred to a polypropylene tube, apportioned into aliquots, and stored at −20°C.

### Peptides.

Peptides for HERV-W-env_(248–262)_ (derived from the HERV-W-env surface protein), IFN-α, and IFN-ω were designed using the Immuno Epitope Database and Analysis Resource (IEBD) and synthesized at >95% purity (LifeTein, South Plainfield, NJ, USA). IEDB software predicts regions of proteins that are likely to be recognized as epitopes in the context of a B cell response. All peptides were dissolved in dimethyl sulfoxide (DMSO) and stored at −80°C in single-use aliquots (10 mM) (Table 1).

### Enzyme-linked immunosorbent assay.

The samples were tested for the presence of Abs against HERV-W_(248–262)_, IFN-α, and IFN-ω antigens using an indirect enzyme-linked immunosorbent assay (ELISA). Ninety-six-well Nunc immunoplates were incubated overnight at 4°C with 10 μg/mL of the respective peptides in 0.05 M carbonate-bicarbonate (pH 9.5) (Sigma-Aldrich, St. Louis, MO, USA).

Plates were washed with 0.1% Tween 20 in phosphate-buffered saline (PBS-T) and blocked with a solution of 5% skimmed milk for 1 h at room temperature. Plasma samples, diluted 1:100, were added, and the plates were incubated for 2 h. After another washing step, the plates were incubated for 1 h with 100 μL of PBS and an alkaline phosphatase-conjugated goat anti-human IgG polyclonal antibody (Sigma-Aldrich, St. Louis, MO, USA). Alkaline phosphatase was detected after 8 min of incubation in a solution containing MilliQ water and *para*-nitrophenyl phosphate (Sigma). The optical density was read at 405 nm using the SpectraMax Plus 384 system (Molecular Devices, Sunnyvale, CA, USA).

Each sample was run in duplicate. Positive- and negative-control plasma samples were also included in all experiments.

A competitive ELISA was performed by using IFN-α, IFN-ω, and a different peptide (annexin A2 positions 13 to 37, LEGDHSTPPSAYGSVKAYTNFDAER, to which the patient was positive) in order to assess the specificity of binding for each peptide.

### Statistical analysis.

Statistical analysis was performed using GraphPad Prism 8.2.0 software (GraphPad, San Diego, CA, USA). Kruskal-Wallis and Dunn’s *post hoc* tests were carried out to compare differences among the HC, COVID-19, and ICU groups. Statistically significant differences were set at a *P* value of <0.05. A Spearman correlation test was performed among the levels of Abs to HERV-W_(248–262)_- and IFN-derived peptides.
